# A Focus on the Nowadays Potential Antiviral Strategies in Early Phase of Coronavirus Disease 2019 (Covid-19): A Narrative Review

**DOI:** 10.3390/life10080146

**Published:** 2020-08-09

**Authors:** Caterina Monari, Valeria Gentile, Clarissa Camaioni, Giulia Marino, Nicola Coppola, Vanvitelli COVID-19 group

**Affiliations:** Department of Mental Health and Public Medicine—Infectious Diseases Unit, University of Campania Luigi Vanvitelli, 81100 Naples, Italy; caterina.monari@gmail.com (C.M.); valeria.gentile25@gmail.com (V.G.); clarissacamaioni91@gmail.com (C.C.); giulia.marino_a@tiscali.it (G.M.)

**Keywords:** COVID-19, antiviral therapy, early phase, current evidences, current recommendations, management

## Abstract

**Background**: The outbreak of the severe acute respiratory syndrome coronavirus 2 (SARS-CoV-2) infection and the related disease (COVID-19) has rapidly spread to a pandemic proportion, increasing the demands on health systems for the containment and management of COVID-19. Nowadays, one of the critical issues still to be pointed out regards COVID-19 treatment regimens and timing: which drug, in which phase, for how long? **Methods**: Our narrative review, developed using MEDLINE and EMBASE, summarizes the main evidences in favor or against the current proposed treatment regimens for COVID-19, with a particular focus on antiviral agents. Results: Although many agents have been proposed as possible treatment, to date, any of the potential drugs against SARS-CoV-2 has shown to be safe and effective for treating COVID-19. Despite the lack of definitive evidence, remdesivir remains the only antiviral with encouraging effects in hospitalized patients with COVID-19. **Conclusions**: In such a complex moment of global health emergency, it is hard to demand scientific evidence. Nevertheless, randomized clinical trials aiming to identify effective and safe drugs against SARS-CoV-2 infection are urgently needed in order to confirm or reject the currently available evidence.

## 1. Introduction

The new coronavirus disease 2019 (COVID-19) caused by severe acute respiratory syndrome coronavirus-2 (SARS-CoV-2) began in Wuhan, China, in December 2019 [1]. Since then, it has rapidly spread worldwide, so far that in March 2020, the World Health Organization (WHO) has declared it a global pandemic and public health emergency [2]. By 1st August 2020, the WHO has reported a total of 17,106,007 confirmed cases and 668,910 confirmed deaths due to COVID-19 globally [3].

The novel coronavirus is structurally similar to severe acute respiratory syndrome coronavirus-1 (SARS-CoV-1) and Middle East respiratory syndrome coronavirus (MERS), which were responsible for the outbreaks in 2003 and 2013, respectively [1]. Considering the viral phylogenetic analogies, the previous experience with SARS and MERS infections provided valuable insights into potential pharmacological therapy for the ongoing pandemic [4,5]. Although several drugs have been proposed as possible COVID-19 treatment, nowadays, none of them has been proved to be safe and effective against SARS-CoV-2. Therefore, there is an urgent need for identifying optimal pharmacological therapies for this disease.

## 2. Viral Cycle Pathogenesis

SARS-CoV-2 is an enveloped, single-stranded RNA beta-coronavirus that is able to enter the host cells through the binding between the viral structural spike (S) protein and the angiotensin-converting enzyme 2 (ACE2) receptor [5]. Viral entry is facilitated by a type 2 transmembrane serine protease, TMPRSS2, via the S protein as well [4]. Once the binding S protein receptor is established, the virus particle enters the host cell through membrane fusion and endocytosis. Inside the cell, the viral genome is released and translated into viral polypeptides, which are then cleaved into small products by proteases. The following stages include RNA synthesis by RNA-dependent RNA polymerase (RdRp), structural proteins synthesis and exocytosis, and release of the new assembled virions [5].

Thus, the possible targets for an effective therapy against COVID-19 are the viral entry in host cells through the S protein-ACE2 receptor and/or TMPRSS2 binding, the interruption of viral polyproteins production by the inhibition of viral protease, and, lastly, viral replication through the inhibition of RdRp.

## 3. Natural History and Proposed Clinical Staging

The natural history of COVID-19 is extremely variable, ranging from asymptomatic infection to pneumonia and, in some cases, even to fatal complications.

According to the viral activity and the host inflammatory response, a three-stage classification system with increasing clinical severity has been proposed [6].

The “early infection” corresponds to the incubation period and initial presentation of the disease. It is characterized by viral entry in human cells through the ACE2 receptors, which are particularly abundant in the lungs, but also in the small intestine epithelium and on the vascular endothelium. At this stage, COVID-19 usually presents with upper airways (cough, sore throat) and systemic symptoms, such as fever, fatigue, conjunctivitis, muscle ache, and less frequently nausea or diarrhea [6]. Symptoms usually appear on average 5 to 6 days after infection, with a 95% confidence interval (CI) ranging from 2 to 14 days [7].

The second stage is characterized by viral replication and pulmonary involvement, usually resulting in a viral pneumonia with bilateral infiltrates or ground glass opacities. During this phase, dyspnoea and alterations in gas exchange, defined as PaO_2_/FiO_2_ < 300 mmHg, until a state of hypoxemia may appear, as well an increase in systemic inflammatory markers [6].

Lastly, a minority of patients progress to the third stage of the disease. This phase is identified by a systemic hyper-inflammation syndrome, with pulmonary and extra-pulmonary symptoms caused by a “cytokine-storm”, which may lead, in most severe cases, to multi-organ failure and shock. In particular, dyspnoea and hypoxia are the most frequent signs of respiratory failure or acute respiratory distress syndrome (ARDS). Inflammatory biomarkers, such as C reactive protein, ferritin, and d-dimer, and cytokines [interleukin-2 (IL2), IL6, IL7, TNFα, granulocyte-colony stimulating) are significantly increased, especially in those patients with more severe disease [8].

## 4. Aim of the Narrative Review

This narrative review will summarize the main therapeutic agents proposed for COVID-19 treatment, with a special focus on the first phase of infection. The article is addressed particularly to physicians taking care of patients with COVID-19 in their clinical practice, in order to identify the best management in the early phase of infection.

## 5. Materials and Methods

A narrative review was performed using MEDLINE, Google Scholar, and Embase from January 2020 up to the beginning of July 2020, in order to identify the main evidences about COVID-19 treatment in the first phase of the infection. The last research was made on the 4 July 2020. We included the following search terms: “COVID-19” and “SARS-CoV-2” in combination with “treatment” and “therapy”. The reference lists of all the included studies were analyzed, in order to identify any other studies that might have deserved inclusion. We excluded the non-English-language articles.

Moreover, we have identified ongoing clinical trials (RCTs) using the search term “COVID-19” and “treatment” or “therapy” on ClinicalTrials.gov.

## 6. Antiviral Agents

The above-mentioned viral life-cycle steps identify potential targets for antiviral drugs, whereas the clinical disease staging may help clinicians to understand the best timing of the different treatment approaches.

The first phase of the disease, i.e., the “early infection”, is characterized by viral entry and viral replication; thus, antivirals may play a key role in the treatment of the SARS-CoV-2 infection.

According to this, many drug targets have been identified. Several agents with apparent in vitro and in vivo activity against SARS-CoV-1 and MERS-CoV have been suggested as potential candidates for SARS-CoV-2, even though the clinical benefits of any of this regimen were not demonstrated.

**Figure 1:**
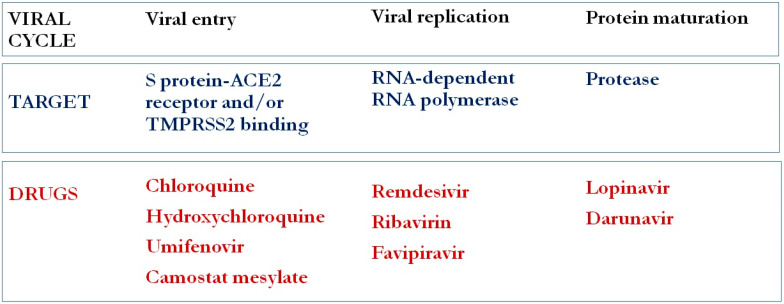
Viral cycle steps and possible therapeutic targets.

In this paragraph, we will review the current evidence regarding the main proposed antiviral drugs for COVID-19 in the first phase of infection. Figure 1 summarizes the viral cycle steps and possible therapeutic targets.

Antiviral agents proposed against SARS-CoV-2, their mechanism of action and their main adverse reactions are summarized in Table 1.

### 6.1. Chloroquine and Hydroxychloroquine

Chloroquine (CQ) and its analogue hydroxychloroquine (HCQ) are drugs that have been used in the past 70 years to treat malaria and, more recently, chronic inflammatory diseases, such as rheumatoid arthritis (RA) and systemic lupus erythematosus (SLE).

CQ and HCQ have similar pharmacokinetic characteristics, with rapid gastrointestinal absorption and renal elimination. The current dose of HCQ sulfate used against COVID-19 consists in a loading dose of 400 mg twice daily for day 1 followed by a maintenance dose of 200 mg twice daily for 4 days, while the one of chloroquine phosphate is 500 mg twice daily orally [10].

The rationale in the use of both chloroquine and hydroxychloroquine in SARS-CoV-2 infection is based on the fact that they seem to hinder viral entry into host cells through the inhibition of glycosylation of host receptors, of proteolytic processing, and of endosomal acidification. Moreover, they have an immunomodulatory effect, secondary to the reduction in cytokine production and an inhibition of autophagy and lysosomal activity in host cells [5,11,12].

However, there are no high-quality evidence supporting the efficacy of HCQ or CQ therapy against SARS or MERS infections [13,14].

Maissonasse et at. evaluated the antiviral activity of HCQ both in vitro and in SARS-CoV-2-infected macaques. In vitro, post-infection treatment of Vero E6 cells with HCQ resulted in a dose-dependent antiviral effect, with 50% inhibitory concentration (IC50) values of 2.2 µM (0.7 µg/mL) and 4.4 µM (1.4 µg/mL) at 48 and 72 h post infection. HCQ tested in vivo in macaques did not show a significant effect on the viral load levels neither alone nor in combination with azithromicin, regardless of the timing of treatment initiation, either before infection, early after infection (before viral load peak), or late after infection (after viral load peak). This study shows a discrepancy from in vitro classic assays and in vivo experiments [15].

Nevertheless, the activity of CQ and HCQ has been further investigated in vitro against SARS-CoV-2, showing that both these drugs decrease the viral replication in a concentration-dependent manner [10]. Moreover, chloroquine shows effectiveness at an entry and post-entry level, suggesting the possible prophylactic and therapeutic activity of this molecule against SARS-CoV-2 [16].

Given these promising in vitro results and the scenario of global emergency, several clinical trials have been launched in order to gather clinical evidence to support the use of these two drugs in COVID19 treatment.

An open-label non-randomized French study reported a better virologic clearance in patients treated with HCQ: the virologic clearance by nasopharyngeal swab for SARS-CoV-2 at day 6 was 70% in 20 patients treated with HCQ compared to 12.5% in the control group (16 subjects). Moreover, the combination of azithromycin and HCQ yielded a higher viral clearance compared to HCQ alone (*p* < 0.05) [17]. In a following study, the same authors confirmed that the use of HCQ plus azithromycin improved clinical outcome in 80 patients, although in absence of a control group of patients [18].

Another study carried out in China on more than 100 COVID-19 patients has shown that CQ was more effective than the control group in enhancing viral clearance, improving imaging findings, and shortening the duration of symptoms [19].

In a randomized trial conducted in Wuhan, China, 62 patients were randomly treated with a 5-day course of HCQ or standard of care: a faster mean time to clinical recovery, i.e., resolution of fever and cough, and an improvement on chest radiography was observed in the experimental group; only 4 patients, all in the control group, showed a progression to severe infection [20].

A recent observational study conducted in New York City retrospectively compared 811 hospitalized patients who received HCQ (600 mg twice on day 1, then 400 mg daily for a median of 5 days) with 565 patients who did not: no significant association between HCQ and lowered risk of intubation or death (HR 1.04; 95% CI 0.82–1.32) was found [21]. However, it should be underlined that patients receiving HCQ were more severely ill at baseline than those in the control group

Arshad et al., taking as the primary outcome the in-hospital mortality, conducted a comparative retrospective cohort study of 2541 hospitalized patients with COVID-19 treated with different therapeutical strategies. Overall in-hospital mortality was 18.1%, 13.5% [95% CI: 11.6%–15.5%] in the hydroxychloroquine only group, 20.1% [95% CI: 17.3%–23.0%] among those with hydroxychloroquine + azithromycin, 22.4% [95% CI: 16.0%–30.1%] among the azithromycin-only group, and 26.4% with neither drug (*p* < 0.001). Treatment with hydroxychloroquine alone and in combination with azithromycin seems associated with a reduction in COVID-19-associated mortality, although the study has many limitations, including its design [22].

A recent randomized double-blind placebo-controlled trial was conducted using oral HCQ or masked placebo in an outpatient who had early, mild COVID-19 or probable COVID-19 and high-risk exposure within 4 days of symptom onset. Among 491 outpatients at 14 days, 24% (49 of 201) of participants receiving hydroxychloroquine had ongoing symptoms compared with 30% (59 of 194) receiving placebo (*P* = 0.21), proving that HCQ did not reduce symptom severity over 14 days [23].

Lastly, a multinational registry analysis has shown a higher mortality rate and an increased risk of ex novo ventricular arrhythmia appearance among patients affected by COVID-19 treated with CQ and HCQ, alone or in combination with a macrolide [24]. However, since these results have raised several concerns, the paper has been retracted by the auhors [25].

Furthermore, in two large randomized control trials, the Solidarity trial by the WHO [26] and Recovery trial by the Oxford University in UK [27], the HCQ arm has been recently ceased because of a lack of its efficacy in a cohort of hospitalized patients with COVID-19. The rationale of this decision is explained in the chapter “ongoing clinical trials”.

As well the DisCoVeRy trial, a multicenter, adaptive, randomized open clinical trial, aiming to evaluate the clinical efficacy and safety of 4 treatment arms (remdesivir, lopinavir/ritonavir (LPV/r), Interferon-beta 1A, HCQ) in addition to the usual standard of care, has temporarily stopped the HCQ arm since 24 May 2020 [28].

Nevertheless, hydroxychloroquine and chloroquine are considered relatively safe and well tolerated. The most common side effects include gastrointestinal symptoms, such as nausea and diarrhea, pruritus, and dermatological alterations. However, both drugs can cause severe side effects (<10%), such as cardiotoxicity, proximal muscles neuromyopathy, hypoglycemia, and retinopathy. In particular, cardiotoxicity can include QT prolongation and arrhythmias, especially in patients with previous renal or hepatic problems [29]. Therefore, an electrocardiography (ECG) is deemed necessary prior to the initiation and during the treatment, especially in those patients taking concomitant QT-interval prolonging drugs, such as azithromycin.

In conclusion, since there is a low level of evidence of CQ and HCQ efficacy against SARS-CoV-2, other clinical trials are needed to clarify the efficacy of HCQ and CQ in COVID-19 treatment and their safety profile.

### 6.2. Lopinavir/Ritonavir (LPV/r) and Other Protease Inhibitors (PIs)

Lopinavir/ritonavir (LPV/r) is an oral combination agent approved for the treatment of HIV infection. Lopinavir is a 1st generation protease inhibitor, whereas ritonavir acts as a booster of LPV by inhibiting cytochrome P450 and P-glycoprotein.

Studies in vitro have demonstrated an antiviral activity of LPV against SARS-CoV-1, MERS-CoV, and other coronaviruses through the inhibition of 3-chymotrypsin-like protease [30,31,32,33]. Moreover, a recent study in vitro has demonstrated the antiviral effect of LPV against SARS-CoV-2 [34]

Clinical studies regarding LPV/r activity against human coronaviruses are few and have been conducted mostly on SARS-CoV-1 infection, with promising results although with retrospective and observational designs [33,35]. For example, a multicenter retrospective matched cohort study including 1052 SARS-infected patients, 75 treated with LPV/r and ribavirin and 977 controls, suggested that the combination therapy was effective against SARS-CoV, in particular in the early phase of infection [35].

Data regarding LPV/r activity against SARS-CoV-2 mostly derive from case reports or small non-randomized, retrospective studies, with controversial results [36,37,38]; therefore, they do not allow asserting the direct efficacy of LPV/r against SARS-CoV-2 [39].

Recently, a randomized, controlled, open-label trial comparing the efficacy of LPV/r versus standard of care was conducted in 199 hospitalized adult patients with severe COVID-19: no significant difference between the two groups neither in the time of clinical improvement (hazard ratio [HR] 1.31; 95% CI 0.95–1.80; p 0.09), nor in the 28-day mortality rate (19.2% versus 25.0%; 95% CI −17.3 to 5.7) was observed [40]. It is worthy of note that in both groups, LPV/r was started late in the course of disease, at a median time of 13 days from the onset of symptoms (interquantile range, IQR 11–16). Thus, the timing of administration of antiviral agents seems crucial: the initiation of LPV/r beyond the peak viral replication phase (initial 7–10 days) had no effect on clinical outcomes [34,35].

RCTs are underway in order to better describe the role of LPV/r in SARS-CoV-2 infection, especially in the early phase.

In particular, the DisCoVeRy trial, a multicenter, adaptive, randomized open clinical trial, aiming to evaluate the clinical efficacy and safety of 4 treatment arms (remdesivir, LPV/r, Interferon-beta 1A, HCQ) in addition to the usual standard of care is still ongoing [28].

However, the Recovery trial by the Oxford University in the UK has recently described no clinical benefit from the use of LPV/r in hospitalized patients with COVID-19 [41]. As a matter of fact, colleagues found no significant difference in the 28-day mortality between 1596 patients treated with LPV/r and 3376 patients randomized to usual care alone (22.1% LPV/r versus 21.3% usual care) nor in the risk of progression to mechanical ventilation or length of hospital stay [41]. However, they were unable to study a large number of patients on mechanical ventilation; therefore, these results may not be applied to severe patients with COVID-19 requiring invasive ventilation.

Other protease inhibitors (PI), such as darunavir/cobicistat (DRV/c) or darunavir/ritonavir (DRV/r), have been identified as potential agents with activity against SARS-CoV-2 infection, thanks to its structural similarity to LPV/r [42]. In fact, in vitro cell models have demonstrated a significant activity of DRV/c against SARS-CoV-2 [42].

However, currently, there are very few data regarding the efficacy and safety profile of DRV/c in COVID-19 patients.

Interestingly, a recent case report has provided preliminary evidence that darunavir did not prevent SARS-CoV-2 infection in three HIV-positive subjects who were assuming DRV/c as part of the antiretroviral regimen [43].

Anyway, clinicians should consider the possible adverse events related to the use of PIs in the treatment for COVID-19. The RCT of Cao et al. showed that adverse events were observed in 50% of patients, thus leading to a drug discontinuation in 14% of them [40]. The most commonly reported adverse effects of LPV/r include gastrointestinal symptoms (up to 28%), such as diarrhea, nausea, and vomiting, hepatotoxicity (2–10%), hypertriglyceridemia, and hypercholesterolemia. Serious adverse reactions have been described as well, including pancreatitis, QT interval prolongation, PR interval prolongation, diabetes mellitus, and/or hyperglycaemia [44]. Thus, an ECG that aimed to study QT intervals should be done prior to and during the treatment, especially in those patients taking concomitant QT-interval prolonging drugs, such as hydroxycholoroquine, chloroquine, or azithromycin.

Lastly, it is important to rule out drug–drug interactions, considering that these agents are CYP3A inhibitors [44].

In conclusion, further studies regarding the efficacy of PIs against SARS-CoV-2 are needed, especially to evaluate the efficacy in the early phase of infection.

### 6.3. Remdesivir

Remdesivir, known as GS-5734, is a novel nucleotide analogue that is the inhibitor of the RNA polymerase. It is a monophosphoramide pro-drug that mimics adenosine, causing the premature termination of viral RNA replication by the inhibition of RdRp. It was originally developed against Ebola virus and it has been proved to have activity against MERS-CoV and SARS-CoV-1 both in vitro and in human cells [45].

In a murine lung infection model, remdesivir showed both prophylactic and therapeutic efficacy against SARS-CoV-1, resulting in a significantly reduced lung viral load and improved clinical signs of disease as well as lung function [46]. Similarly, in a mouse model of MERS-CoV pathogenesis, it improved pulmonary function and reduced lung viral load, both in prophylactic and therapeutic administration, compared to lopinavir/ritonavir and interferon beta [47].

Recently, in vitro data demonstrated that remdesivir had potent antiviral activity against SARS-CoV-2 in Vero cells [16]. Moreover, it exerted significant antiviral and clinical effects in a non-lethal rhesus macaque model [48] and was a potent inhibitor of viral replication in human nasal and bronchial airway epithelial cells [49].

At this time, it is unknown how the observed efficacy of remdesivir against SARS-CoV-2 infection in animal models will translate into clinical efficacy in patients in clinical practice. However, remdesivir has been globally used in hundreds of patients infected with SARS-CoV-2 under a compassionate use protocol or expanded access. In several case series of COVID-19, the use of remdesivir was associated with an improvement in clinical condition [50,51].

In one multicenter, multinational series, 53 patients with severe COVID-19 received remdesivir for up to 10 days: 36 patients (68%) showed a clinical improvement (decreased requirement for oxygen support or hospital discharge) and of the 30 patients who were mechanically ventilated at baseline 17 (57%) were extubated [52].

A randomized, double-blind, placebo-controlled multicenter trial enrolled 236 patients with moderate COVID-19, 155 of whom received remdesivir: although remdesivir was associated with a faster time to clinical improvement (but not significant at statistical analysis), the time to clinical improvement was 21 days in remdesivir group and 23 days in the placebo one (HR 1.23; 95% CI 0.87–1.75), and no difference in 28-days mortality was observed between the two groups [53].

However, the preliminary report of another double-blind, randomized, placebo-controlled trial has been recently published showing encouraging effects of remdesivir in hospitalized adults affected by COVID-19 with involvement of the lower respiratory tract [54]. Of the total 1059 patients, 538 were assigned to the remdesivir group, and 521 were assigned to placebo. The study arm showed a lower median recovery time (11 versus 15 days, *p* < 0.001) and a trend toward lower mortality, although it was not significant (7.1 versus 11.9%).

Another ongoing RCT evaluating the efficacy and safety of remdesivir is the DisCoVeRy trial [28].

On 1 May 2020, the U.S. Food and Drug Administration (FDA) has authorized the emergency use of remdesivir, stating that it can be used to treat “in-hospital adults and children with suspected or laboratory confirmed COVID-19 and severe disease defined as SpO2 ≤ 94% on room air, requiring supplemental oxygen, mechanical ventilation or extracorporeal membrane oxygenation (ECMO)” [55].

The current dose under investigation is an intravenous (i.v.) 200 mg-loading dose on day 1, followed by i.v. 100 mg once daily for a total duration of 5 to 10 days. A recent randomized, open-label, phase 3 trial did not showed a significant difference between a 5-day or a 10-day-course of remdesivir in 397 patients with severe COVID-19 [56].

The safety profile has not been currently established. The most common adverse reactions reported are gastrointestinal (nausea, vomiting, and transient elevation of serum alanine aminotransferase and aspartate aminotransferase), prolonged mild and reversible prothrombin time, and renal toxicity due to the accumulation of sulfobutyl ether β-cyclodextrin sodium (SBECD). In fact, remdesivir is not recommended in case of renal impairment, with an estimated glomerular filtration rate less than 30 mL/min [9].

### 6.4. Other Antivirals

Umifenovir (also known as arbidol) is a drug approved in Russia and in China for oral treatment and prophylaxis of Influenza A and B viruses. It prevents viral entry in the host cell by inhibiting the membrane fusion of the viral envelope and the host cell cytoplasmic membrane [57]. Studies in vitro demonstrated a broad-spectrum antiviral activity against hepatitis B and C viruses, Ebola virus, Lassa virus, Human Herpes virus 8, and poliovirus [58]. Since some data in vitro suggested an antiviral activity against SARS virus, this agent has gained increasing interest as a potential drug for COVID-19. In a single-center retrospective cohort study, 16 patients with COVID-19 receiving umifenovir 200 mg every 8 h plus LPV/r were compared to a control group of 17 patients receiving only LPV/r: compared to the control group, the experimental group showed a higher rate of viral clearance and a more significant improvement in chest imaging [59]. A higher rate of viral clearance due to umifenovir administration was suggested by Zhu et al. as well [60]. An improvement in discharging and the mortality rate was also described in a retrospective study on 67 patients in Wuhan [61]. On the other hand, a Chinese retrospective study, comparing 45 patients in the umifenovir group and 36 in the control group, showed that umifenovir did not improve the prognosis of the patient nor accelerate SARS-CoV-2 clearance [62]. Since these contrasting data, RCTs are underway in China.

Favipiravir (T-705) is an antiviral agent that selectively and potently inhibits the RNA-dependent RNA polymerase (RdRp) of RNA viruses. It is effective against several RNA viruses, including Influenza virus, flavi-, alpha-, filo-, arena- and noro-viruses, as well as West Nile virus, Yellow Fever virus, Ebola virus, and Lassa virus [57,63]. Favipiravir is one of the potential candidates for COVID-19 treatment, although in vitro studies showed controversial results [16,34,64].

A open-label non-randomized study compared the effect of favipiravir to LPV/r in a cohort of 80 patients with COVID-19: favipiravir was associated with a shorter viral clearance time (4 versus 11 days, *p* < 0.001) and significant improvements in chest imaging (91.4% versus 62.2%, p 0.004) [65]. Clinical trials aiming to describe the efficacy of favipiravir *against COVID-19 are underway*.

Camostat mesylate is an agent approved in Japan for the treatment of pancreatitis. It is a serine protease inhibitor, which seems to prevent SARS-CoV-2 entry in human lung cells through inhibition of the host serine protease TMPRSS2 [66]. Gabexate mesylate and nafamostat mesylate are similar agents and have been studied against COVID-19 as well. In vitro studies demonstrated a promising effect of all these agents in inhibiting SARS-CoV-2, in particular nafamostat mesylate [67]. Nevertheless, further evaluations in clinical trials are needed.

Ribavirin is a guanine analogue that inhibits viral RNA-dependent RNA polymerase. It is used to treat several virus infections, such as respiratory syncytial virus (RSV), hepatitis C virus, and some viral hemorrhagic fevers. Moreover, it demonstrated activity against other novel coronaviruses, making it a candidate for COVID-19 treatment. Although promising results were previously obtained with ribavirin and IFN-alfa 2b in a MERS-CoV rhesus macaque model [68], in human MERS infection, data have been discordant [69]. In SARS-CoV-1, a systematic review showed inconclusive results in 26 of 30 studies, with 4 of them showing possible harms due to adverse reactions [70].

Interferon (IFN) both interferes with viral replication and regulates the immune system. It is an antiviral drug used in both hepatitis B and C virus infection, and it has been seen to have efficacy in reducing viral replication and disease severity in animal models of MERS infection, particularly when used in combination with other drugs [71,72,73]. In contrast, a MERS-CoV infection model of humanized transgenic mouse demonstrated that the combination of IFN-beta and LPV/r improved pulmonary function but did not reduce virus replication or severe lung pathology [47]. Currently, as a result of the conflicting in vitro and animal data and the lack of clinical trials, the use of interferon cannot be recommended in SARS-CoV-2 infections. The “Solidarity” trial by the WHO is underway and aims to better describe the efficacy of different COVID-19 treatment approaches, including IFN-beta [26]. Another RCT evaluating IFN-beta efficacy and safety profile is the DisCoVeRy trial [28]

Ivermectin is an FDA-approved anti-parasitic agent that showed a broad spectrum antiviral activity in vitro [74], which was probably thanks to the inhibition of nuclear import of viral and host proteins, in particular through the inhibition of importin (IMP) α/β1 [75]. Recently, Caly et al. reported that ivermectin potently inhibited SARS-CoV-2 in Vero/hSLAM cells with a 5000-fold reduction of viral RNA at 48 h [74]. However, its mechanism of action against SARS-CoV-2 is still unclear, and its safety profile has not been established. Moreover, a recent study analyzing its pharmacokinetic profile stated that ivermectin is unlikely to reach the IC50 in lungs after a single standard dose (200 μg/kg), even for a dose 10× higher than the standard one [76]. Therefore, further studies are needed to clarify the efficacy, tolerability, and safety profile of this agent.

However, a recent paper by Stauffer et al. has highlighted the relevant role that ivermectine may play in COVID-19 patients in preventing Strongyloides hyperinfection, which is a potential fatal complication secondary to the administration of dexamethasone [77]. Since there is a high mortality related to this sydrome, the authors propose a test-and-treat strategy and, when it is not possible, they suggest to consider a presumptive treatment with ivermectine in moderate to high-risk patients for Strongyloides [77].

Lastly, the combination of nitazoxanide/azithromycin has been proposed as a potential treatment in the early phase of COVID-19 [78], since the in vitro antiviral activity of nitazoxanide against MERS-CoV and other coronaviruses [79]. However, further studies are deemed necessary to better understand these preliminary results.

## 7. Ongoing Clinical Trials

Nowadays, evidence from RCTs regarding the safety profile and efficacy of the proposed therapies are still lacking. Currently, there are 2890 clinical trials in progress, of which 830 are not recruiting yet. Ongoing clinical trials regarding COVID-19 are available on the website: https://clinicaltrials.gov/ct2/results?cond=COVID-19.

It is worthy of note that the WHO has launched an international multicenter randomized clinical trial, “the Solidarity Trial”, comparing four treatment options (hydroxychloroquine, LPV/r, LPV/r + IFN-β, remdesivir) to standard of care, in order to identify whether any of these drugs are effective in slowing down the disease or improving survival [26]. However, after a review of the interim analysis of the Solidarity trial, including the DisCoVeRy trial data [28], and thanks to the results of other randomized evidences (Recovery trial [27]), the Executive Group has recently decided to withdraw the HCQ arm because of its ineffectiveness in reducing mortality among hospitalized COVID-19 patients [26]. Nevertheless, these results are not applicable to the use of HCQ in non-hospitalized patients, where evidences about effectiveness or ineffectiveness of this drug are still lacking.

The Recovery trial has ceased its HCQ arm for the same reason. Moreover, the Chief Investigator of the Recovery trial has recently decided to close also the randomization to the LPV/r arm because of the lack of beneficial effects in hospitalized patients [41]. Nevertheless, colleagues could not study this drug in a large number of severe patients on mechanical ventilation; therefore, they cannot make conclusions about the efficacy of LPV/r in mechanical ventilated patients.

## 8. Current Recommendations

Table 2 shows the main international scientific societies (WHO, International Society of Infectious Diseases (IDSA), Centers for Disease Control and Prevention (CDC), and National Institute of Health (NIH)) recommendations regarding antiviral treatment regimens for COVID-19. As described above, since there are no evidences from RCTs that any therapy improves the outcome of patients affected by COVID-19, all recommendations highlight that patients should be treated in the context of a formal clinical trial in order to establish drugs safety, efficacy, risks, and benefits. As a matter of fact, antiviral drugs are not free of adverse events, which should be considered and monitored. The most important and frequent adverse events of antiviral drugs used against SARS-CoV-2 are reported in Table 1.

Awaiting the results of the ongoing trials, at the moment, it is only possible to hypothesize a different treatment strategy according to the different phases of COVID-19. In this view, the clinical classification proposed by Siddiqi et al. may guide clinicians in the treatment decision process and identify which is the best therapy for each stage of the disease (Table 3).

In the early phase of infection, the treatment is mainly targeted toward symptomatic relief, while antiviral agents may reduce the duration and progression of symptoms severity. Drugs with antiviral activity seem important in the second stage as well, together with supportive measures, such as early O2 therapy (in case of hypoxia) and anti-inflammatory therapy. Lastly, in the third stage, immunomodulatory agents, such as corticosteroids or cytokine inhibitors (IL6-receptor or IL1-receptor blockers), seem pivotal in order to reduce systemic hyper-inflammation and avoid the progression to a more severe disease.

Therefore, it seems that the sooner antiviral therapy is started, the better its efficacy; whereas its usefulness is uncertain in late stages [16,40]. On the contrary, the use of immunosuppressive agents may not be necessary in early stages, whereas it becomes essential when hyper-inflammation appears.

In conclusion, the rapid identification of the critical situation and the early administration of the above-mentioned therapies are the mainstay for patients outcome [6].

## 9. Conclusions

The COVID-19 pandemic represents the greatest global public health threat of the last decades, with devastating effects not only on public health but also on economic and financial sectors [6,84,85,86]. In this scenario, identifying treatments against SARS-CoV-2 infection effective in the first phase of infection is crucial. In fact, in this phase, an effective antiviral drug may stop the spread of COVID-19 and its progression toward severe forms of disease and may reduce morbidity and mortality.

Many antiviral drugs have been proposed as potential COVID-19 treatment in this first phase. However, at the moment, there is no high-quality evidence to support any of the currently proposed treatment in improving clinical outcomes. As a result of the lack of scientific evidences, COVID-19 treatment remains an issue for clinicians and scientific communities.

In this situation, randomized clinical trials aiming to identify effective and safe drugs against SARS-CoV-2 infection are essential.

## Figures and Tables

**Table 1:** Different treatment regimens, mechanism of action and adverse reactions of main proposed drugs for coronavirus disease 2019 (COVID-19).

Drug Name	Mechanism of Action	Dosing *	Adverse Events	
**Chloroquine (CQ) and Hydroxychloroquine (HCQ)**	- Antiviral activity: by hindering viral entry into host cells through the inhibition of glycosylation of host receptors, proteolytic processing and endosomal acidification - Immunomodulatory effects, with consequent reduction in pro-inflammatory cytokines	- CQ tab 500 mg: 1 tab BID - HCQ tab 200 mg: LD 2 tab BID day1, then 1 tab BID HCQ: long elimination; half-life of 40–55 days	HCQ has a lower incidence of toxicity than CQ. Cardiological: QTc prolongation, torsade de pointes, ventricular arrythmia and cardiac deaths. Baseline and follow-up electrocardiogram (ECG) are recommended especially when coadministered with other QT-interval prolonging drugs [antibiotics (ex. azithromycin), antifungals, antiarrhythmics, antipsychotics] Gastrointestinal: nausea, diarrhea, vomiting Others: hemolysis (if G6PD-deficiency), hypoglycemia, pruritus and dermatological alterations (rash), retinopathy, bone marrow suppression, proximal muscles neuromyopathy, likely with long-term use.	
**Azithromycin**	- Antiviral effects: induction of IFN-stimulated genes, attenuating viral replication - Immunomodulatory effect: enhanced neutrophil activation - Anti-Inflammatory effects: attenuation of inflammatory cytokines (IL-6, IL-8) in epithelial cells and inhibition of fibroblast growth factor in airway smooth muscle cell	tab 500 mg: 1 tab QD	When used with HCQ: Cardiological: QTc prolongation (in particular when coadministered with other QT-interval prolonging drugs) Gastrointestinal: nausea, diarrhea, vomiting Hepatotoxicity	
**HCQ + Azithromycin**	See above			
**HIV Protease Inhibitors** **(LPV/r and DRV/c)**	Possible inhibition of SARS-CoV-2 3-chymotrisyn-like (3CL)-protease and papain-like protease Lopinavir is excreted in the gastrointestinal (GI) tract, and thus coronavirus-infected enterocytes might be exposed to higher concentrations of the drug	LPV/r tab 200/50 mg: 2 tab BID LPV/r oral sol 80/20 mg: 5 mL BID DRV/cobi tab 800/150 mg: 1 tab QD	Gastrointestinal: diarrhea, nausea, vomiting, increased amylase, lipase, total cholesterol and triglycerides (risk factor for pancreatitis) Hepatotoxicity: increasing in GGT, AST, ALT, total bilirubin, hepatitis Cardiological: QT- and PR-interval prolongation, hypertension, bradyarrhytmias; torsade de pointes have been reported in patients treated with LPV/r Metabolical: hyperglycemia and diabetes mellitus, increased uric acid	
**Remdesivir** **(GS-5734)**	Adenosine nucleotide analog prodrug, which inhibits viral RNA-dependent RNA polymerase (RdRp). Potent in vitro activity demonstrated in SARS-CoV-2-infected Vero E6 cells In vitro and in vivo activity vs. SARS-CoV and MERS-CoV	fl 150 mg: LD 200 mg on day 1, then 100 mg QD on days 2-10	Gastrointestinal: nausea, vomiting Hepatotoxicity: transient elevation of ALT and AST (grade 1 or 2), typically after multiple days of treatment [9] Hematological: mild, reversible prolonged prothrombin time (PT) without INR change (Gilead 2020) Renal: potential toxicity due to accumulation of sulfobutyl ether β-cyclodextrin sodium (SBECD) in moderate to severe renal impairment	

* There are no approved doses for the treatment of COVID-19. The doses listed here are for approved indications or from reported experiences or clinical trials for COVID-19. G6PD: Glucose-6-phospate-dehydrogenase; LD: loading dose; QD: quaque die (once a day); BID: bis in die; IFN: interferon; GGT: gamma glutamyl transpeptidase; AST: aspartate aminotransferase, ALT: alanine aminotransferase.

**Table 2:** World Health Organization (WHO), International Society of Infectious Diseases (IDSA), and National Institute of Health (NIH) recommendations regarding COVID-19 treatment regimens [80,81,82,83].

	CQ or HCQ	HCQ + Azithromicyn	LPV/r or Others PIs	Remdesivir
**WHO**	None of the drugs proposed as potential therapies against COVID-19 have been shown to be safe and effective. Many agents are now being or will soon be studied in clinical trials (including the SOLIDARITY trial). “We recommend that the following drugs not be administered as treatment or prophylaxis for COVID-19, outside of the context of clinical trials. … If it is not possible to give the treatment as part of a clinical trial, appropriate records of the use of the medicine must be kept, in compliance with national law, and outcomes for patients should be monitored and recorded. If early results from an unproven or experimental treatment are promising, the treatment should be studied in the context of a formal clinical trial to establish its safety, efficacy, risks, and benefits.”			
**IDSA**	The panel recommends HCQ or CQ in the context of a clinical trial among hospitalized patients with COVID 19	The panel recommends HCQ or CQ + azithromycin only in the context of a clinical trial among hospitalized patients with COVID-19	The panel recommends HCQ or CQ in the context of a clinical trial among hospitalized patients with COVID	/
**NIH**	There are insufficient clinical data to recommend either FOR or AGAINST CQ or HCQ for the treatment of COVID-19 (AIII). When they are used, monitor adverse effects, especially QTc interval prolongation (AIII).	The panel recommends AGAINST the use of HCQ plus azithromycin, except in the context of a clinical trial (AIII).	The panel recommends AGAINST the use of lopinavir/ritonavir (AI) or other HIV protease inhibitors (AIII), except in the context of a clinical trial.	There are insufficient clinical data to recommend either FOR or AGAINST the use of remdesivir for the treatment of COVID-19 (AIII).

**Table 3:** Clinical presentation and treatment strategies in the different phases of COVID-19.

	Early (Viral) Phase	Intermediate (Pulmonary) Phase	Late (Cytokine Storm) Phase
**Pathogenesis**	Viral entry in host cells (S protein-ACE2 or—TMPRSS2 binding)	Viral replication, mainly in lower respiratory airways	Systemic hyper-inflammation syndrome and cytokine-storm, mainly in the lung
**Clinical presentation**	Mild upper airways symptoms: dry cough, sore throat, conjunctivitis, malaise ±hypo- or a-nosmia ±hypo- or a-geusia Mild constitutional symptoms: fever, headache, muscle ache and less frequently nausea or diarrhea	Fever (recurrent or persistent) Lower respiratory tract involvement (viral pneumonia) In late phase, possible dyspnoea, shortness of breath and hypoxia ±extra-pulmonary symptoms	ARDS Shock ±Multi-organ failure may appear in most severe cases
**Biochemical presentation**	Leucopenia with lymphopenia, Mild increase in: prothrombin time, D-dimer, C reactive protein (CRP) and LDH	Lymphopenia Increase in: ALT, AST Increased systemic inflammatory markers: CRP, ferritin, LDH, D-dimer, IL-6 In late phase, possible hypoxemia	Lymphopenia Significant increased systemic inflammatory markers: CRP, ferritin, LDH, D-dimer, cytokines (IL2, IL6, IL7, TNFα, granulocyte-colony stimulating, etc.), procalcitonin Increase in: troponin, NT-proBNP, ALT, AST Severe hypoxemia
**Imaging (RX, Tc) signs**	None	Pneumonia with bilateral ground glass opacities (GGO) and/or consolidations	Increasing in number, dimension and density of bilateral consolidations until ARDS scenario
**Treatment strategy**	- Symptomatic therapy - Antiviral agents	- Supportive therapy (O_2_ therapy) - Antiviral agents - Heparin - ±Antiinflammatory/Immunomodulatory agents: corticosteroids and/or Interleukin inhibitors	- Supportive therapy (non-invasive or invasive O_2_ mechanical ventilation) - Antiviral agents (remdesivir) - Heparin - Antinflammatory/Immunomodulatory agents: corticosteroids and Interleukin inhibitors
